# A New Journal

**Published:** 1881-12

**Authors:** 


### A New Journal.

We are glad to welcome to our list of exchanges still another journal—this one being named the *New England Medical Monthly*. If future numbers are equal to the first one, it must certainly prove a success. Professor Post, of New York, furnishes the opening article; another is contributed by Professor Carmalt, of Yale College, and similarly excellent papers, together with sprightly editorials, combine to make the first impressions of this new-comer in the field of journalism most agreeable.

